# Causal Relationships between Daily Physical Activity, Physical Function, and Cognitive Function Ultimately Leading to Happiness

**DOI:** 10.3390/ijerph20043016

**Published:** 2023-02-09

**Authors:** Yuna Inada, Chihiro Tohda

**Affiliations:** Section of Neuromedical Science, Division of Bioscience, Institute of Natural Medicine, University of Toyama, Toyama 930-0194, Japan

**Keywords:** physical health, subjective mental health, happiness, quality of life, structural equation modeling

## Abstract

Frailty is a common age-related condition linked with mobility disorders, long-term care, and death. To prevent frailty, physical activities are considered effective. Several studies have indicated that physical activity can influence mental health as well as body function. Physical activity, cognitive function, and subjective mental health must relate to each other. However, most studies only focus on one-to-one interactions. This observational study aims to clarify the overall relationship and causality between subjective mental health, daily physical activity, and physical and cognitive functions. We recruited 45 people (24 males and 21 females) over 65 years old. Participants visited the university twice and were subjected to activity measurements at home. To examine the causal relationships and related structures between the indicators, structural equation modeling was performed. The results suggest that daily physical activity explains physical function, physical function explains cognitive function, and cognitive function explains subjective mental health, quality of life, and happiness. This study is the first to clarify interactive relationships as an axis that start from daily physical activity to happiness in older adults. Upregulating daily physical activity may improve physical and cognitive functions as well as mental health; this might protect and ameliorate physical, mental, and social frailties.

## 1. Introduction

Frailty is a common age-related condition linked with mobility disorders, long-term care, and death. Frailty is characterized by multiple aspects, such as physical, psychological, and social frailty. Sarcopenia is defined as one of the statuses of physical frailty. Several studies suggest that physical activity probably prevents frailties, such as weight loss, exhaustion, and slow walking speed [1].

Very interestingly, studies indicating that physical activity not only influences body function, but also neurofunction. Physical exercise three times a week for six months improves cognitive function in healthy older adults [2]. In randomized trials for an old person with subjective memory impairment, six-month physical activity programs were effective in improving cognitive function and the effects lasted for 12 months [3]. Short-term (five to seven days) exercise also improved cognitive function in hospitalized, frail, older adult patients [4]. In a systematic review and meta-analysis of randomized controlled trials, aerobic exercise was effective in improving cognitive function in people with Alzheimer’s disease [5]. Taken together, regardless of the level of one’s cognitive function, meta-analyses have shown that physical exercise improves cognitive function [6]. Accumulating reports indicate that elevated levels of insulin-like growth factor, brain-derived neurotrophic factor (BDNF), and other neurotrophic factors are involved in the improvement of cognitive function through exercise [7,8,9,10,11]. Meanwhile, an 18-month exercise intervention did not show any cognitive improvement in healthy older adults [12]. Comparing the effects of physical exercise in originally active and inactive older adults, the intervention was not effective on cognitive performance in both groups [13]. In addition, exercise training in patients with dementia did not improve their cognitive function [14]. Therefore, further studies are needed to understand the relationship between exercise intervention and improvement in cognitive function.

While attention has focused on the relationship between physical activity and cognitive function, the effects of physical activity on subjective mental health have also been reported. Eight weeks of exercise training improved the well-being and reduced the physical pain of older adults in need of care and who were suffering from physical pain [15]. A clinical study of 1635 participants randomly assigned to a sedentary and exercise group showed that physical activity intervention slowed the decline in health-related quality of life scores [16]. A large-scale health survey also indicated that leisure-time physical activity was associated with reduced odds of unhappiness after two to four years [17]. These studies suggest that exercise interventions and physical activity in leisure activities may have a positive impact on mental and physical frailty.

Furthermore, the relationship between cognitive function and happiness was also studied. In an analysis of 560 people without dementia, rapid cognitive decline preceded well-being lowering [18]. Another study on healthy older adults demonstrated that a high quality of life was associated with higher cognitive function [19]. As mentioned above, physical activity, cognitive function, and subjective mental health (quality of life and happiness) must relate to each other.

We hypothesize that physical activity significantly contributes to the improvement of cognitive function and well-being. However, most studies just focus on one-to-one interactions. No studies have been performed to clarify the overall interactions with a causality perspective. Uncovering the causal relationship between physical, cognitive, and subjective mental health levels is very important to identify the essential and effective factors or their axis for fulfilling a healthy life. Therefore, this study aims to clarify the relationship and causality between subjective mental health, daily physical activity, physical activity, and cognitive function.

## 2. Materials and Methods

### 2.1. Participants

The inclusion criteria were older adults aged 65 years and older, who could walk independently, climb stairs daily, were not cognitively impaired (Mini-Mental State Examination (MMSE) score of 25 or more), and were unimpaired in their daily lives. All participants were recruited through newspaper flyers distributed in the community and volunteered to participate. They participated from 20 July 2021 to 20 May 2022. This observational study was conducted with the approval of the Ethics Committee of the University of Toyama (R2021024). Each participant signed an informed consent form and took cognitive function tests on test day 1. Subjective mental health questionnaires and physical function tests were evaluated on test day 2. All participants agreed to visit the University of Toyama twice for assessments and wear a triaxial accelerometer device at home for seven days.

### 2.2. Measurement

All tests in this study were conducted by an experienced and well-trained psychologist.

#### 2.2.1. Subjective Mental Health Indicator

To evaluate subjective mental health, happiness, and quality of life (QOL), questionnaires were completed. Happiness was measured by the Oxford Happiness Questionnaire (OHQ) [20]. The OHQ consists of 29 items with a score of 1 (strongly disagree) to 6 (strongly agree). The average of the answers to each of the 29 items was the individual score. The average value of answers to 29 items was the final individual score. The OHQ is less burdensome for participants because the 29 questions are presented in an easy-to-understand one-sentence format. It covers all aspects of well-being with confirmed correlations to extraversion, neuroticism, psychoticism, satisfaction with life, self-esteem, and depression. QOL was measured by the World Health Organization Quality of Life-26 Japanese version (WHOQOL26) [21]. The WHOQOL26 consists of 26 items with a score of 1 (not at all) to 5 (completely). The average value of answers for 26 items was the final individual score. The WHOQOL26 has five subscales designed to provide a detailed QOL assessment and the scale is widely used worldwide. The subjective mental health questionnaires required about 15 min to answer them.

#### 2.2.2. Cognitive Function Indicator

Cognitive function was examined with the Mini-Mental State Examination Japanese version (MMSE-J) and the Wechsler Adult Intelligence Scale—4th ed. (WAIS-IV). The MMSE-J is a dementia screening test with a 30-point scale; it is a tool used to check for the presence of cognitive impairment [22]. In this study, the criterion for the MMSE-J score was over 25 points to include only healthy participants. The WAIS-IV is an intelligence test for adults, consisting of 10 basic and five subscale tests for a full set [23]. The purpose of using WAIS-IV in this study was not to detect cognitive impairment, but to accurately detect a wide range of cognitive function levels from low to high. The WAIS-IV has various domains to evaluate cognitive functions and can help calculate age-appropriate scores. The present study focused on only the seven basic WAIS-IV tests (Block Design, Similarities, Digit Span, Matrix Reasoning, Symbol Search, Information, and Coding) to reduce the participants’ burden. Two cognitive function tests required about 60 min to be performed.

#### 2.2.3. Physical Function Indicator

Physical function was examined using a questionnaire (the 25-question Geriatric Locomotive Function Scale: GLFS-25) and other three tests focusing on lower-limb function (2-step, 5 m walk, and stand-up tests). GLFS-25 is a screening self-reported questionnaire tool to detect locomotive syndrome [24]. It includes 25 items with a score of 0 to 4 for each item: 4 questions regarding pain, 16 questions regarding daily activities, 3 questions regarding social function, and 2 questions regarding subjective mental health status. The score ranges from 0 to 100, with higher scores indicating a poorer condition.

The five-meter walk test assessed walking speed [25] by measuring the time to pass 5 m by walking. On a 6 m long road, the first 1 m was set as an acceleration section, and the following 5 m was an estimated section. The score was calculated as the distance per second as walking speed. Measurements were performed twice, and the fastest walking speed, without running, was recorded.

The two-step test assesses walking ability [26]. The participant starts from a standing posture and is asked to take steps forward with a maximum stride without losing balance. The length of two steps was measured from toe to toe. The score was calculated using the total length of two steps (cm) divided by the participant’s height (cm).

The stand-up test [26] was used to measure lower-limb muscle strength. It evaluates an individual’s ability to stand using both legs, initially, and then with one leg, from a sitting position on stools at heights of 40, 30, 20, and 10 cm. The results are expressed as summed scores, using previously reported scoring methods [27].

All physical function tests required about 30 min to be performed.

#### 2.2.4. Daily Physical Activity Indicator

Participants wore a triaxial accelerometer (GT3X+, Actigraph LLC, Pensacola, FL, USA) at hip level. It was attached to an elastic belt worn around the waist for seven consecutive days to record their daily physical activity level. The device was given to participants on test day 2 or posted to their houses after test day 2. The accelerometer was worn on the right side during all activities, including sleeping time. After 7 days of measurements, participants returned the accelerometers to the examiner, and the examiner output the data of calorie consumption (CC), steps, and moderate-to-vigorous physical activity (MVPA) from each device.

### 2.3. Statistical Analysis

Spearman’s correlation analysis was performed to examine the association between each of the indicators. Structural equation modeling (SEM) was also used to examine the causal relationships and related structures between indicators. In this analysis method, a combination of latent (represented by a circle) and observed (represented by a square) valuables was used. To calculate the sample size, the effect size was set to 0.35 (large), α to 0.05, statistical power (1-β) to 0.8, and predictor to 5 (assuming the number of the path to be drawn in the SEM). Using G*Power software to calculate the power of multiple regression analysis, which is a method similar to SEM analysis, the required sample size was 43. From this, we set the sample size to 45. Statistical analysis was performed using SPSS and AMOS (IBM, Chicago, IL, USA).

## 3. Results

**Figure 1:**
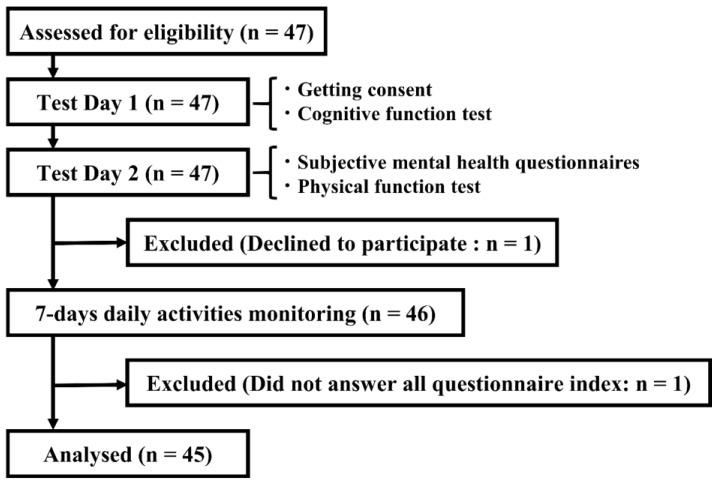
Study flow.

In total, 45 people aged 65 years and over were recruited for this study (Figure 1). The interval from test days 1 to 2 was 44.4 (±40.8) days. The mean age was 72.78 ± 5.23 years, including 24 males and 21 females (Table 1). The MMSE-J and WAIS-IV scores were 28.29 and 87.42, respectively, showing that the cognitive function of participants was at least at a normal or better than normal level. Participants had no history of cognitive or motor dysfunction due to cerebrovascular disease or other causes, and their chronic illnesses were stable at the time of participation. The post hoc power analysis was conducted using G*power software. Setting with an effect size of 0.35, a significance level of 0.05, and a total sample size of 45, the power was 0.87, which was sufficient.

Table 2 shows the correlation of all indicators of subjective mental health, cognitive function, physical function, and daily physical activity. Statistically significant correlations were found within indicators, such as happiness vs. QOL (r = 0.70, *p* = 0.00), MMSE-J vs. WIAIS-IV (r = 0.43, *p* = 0.00), 5 m walk vs. 2-step (r = 0.51, *p* = 0.00), stand up vs. 2-step (r = 0.44, *p* = 0.00), steps vs. calorie consumption (r = 0.78, *p* = 0.00), MVPA vs. calorie consumption (r = 0.83, *p* = 0.00), and MVPA vs. steps (r = 0.83, *p* = 0.00). In addition, statistically significant correlations between indicators were also shown, such as physical function vs. cognitive function, daily physical activity vs. subjective mental health, cognitive function, or physical function.

**Figure 2:**
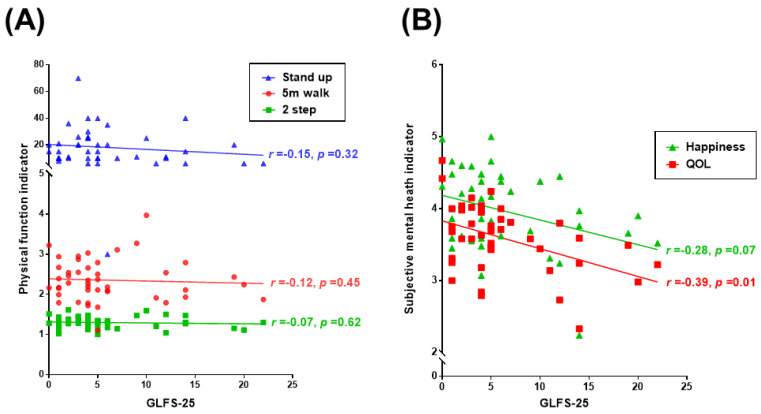

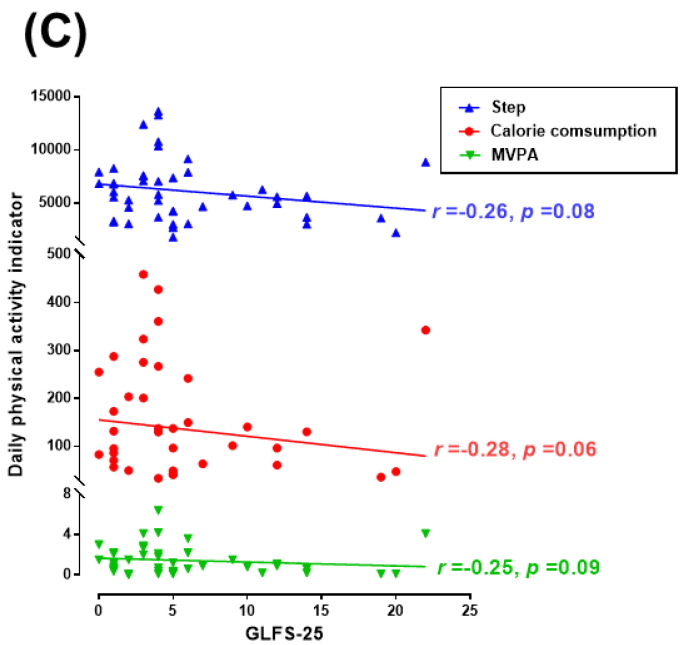
Correlations between GLFS-25 and physical function, subjective mental health, and daily physical activity. (**A**) The relationships between the GLFS-25 and physical function (5 m walk, 2-step, and stand-up tests). (**B**) The relationship between the GLFS-25 and subjective mental health (happiness and QOL). (**C**) The relationships between the GLFS-25 and daily physical activities (calorie consumption, steps, and MVPA). GLFS-25: 25-Question Geriatric Locomotive Function Scale; QOL: WHOQOL26; Happiness: Oxford Happiness Questionnaire; and MVPA: Moderate-to-Vigorous Physical Activity.

GLFS-25, a questionnaire about physical disability in daily life is one of the tests for physical function. Figure 2A shows the relationship between the GLFS-25 and physical function (5 m walk, 2-step, and stand-up tests). There was no statistically significant correlation. Figure 2B shows the relationship between the GLFS-25 and subjective mental health (happiness and QOL). Figure 2C shows the relationship between the GLFS-25 and daily physical activities. The GLFS-25 was negatively associated with happiness and QOL (happiness r = −0.28, *p* = 0.07; QOL r = −0.39, *p* = 0.01), and the GLFS-25 was also associated with daily physical activities (calorie consumption r = −0.28, *p* = 0.06; steps r = −0.26, *p* = 0.08; MVPA r = −0.25, *p* = 0.09).

**Figure 3:**
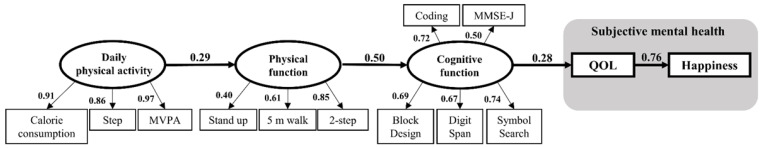
The SEM model predicts the association of subjective mental health, cognitive function, physical function, and daily physical activity indicators. Latent variables are represented by circles and observed variables by squares. The numbers represent path coefficients (β). The goodness-of-fit values of the SEM are GFI = 0.800, AGFI = 0.712, CFI = 0.946, and RMSEA = 0.070. All path coefficients are significant or show a statistically significant tendency in this model. MVPA: Moderate-to-Vigorous Physical Activity; MMSE-J: Mini-Mental State Examination-Japanese version; QOL: WHOQOL26; and Happiness: Oxford Happiness Questionnaire.

Figure 3 shows the SEM model that predicts the association of subjective mental health, cognitive function, physical function, and daily physical activity indicators. Standardized coefficients displayed above the arrows represent direct associations between the variables. The results of SEM indicate that daily physical activity explains physical function; physical function explains cognitive function; cognitive function explains QOL; and, finally, QOL leads to happiness. A single axis can be drawn from daily physical activity to happiness (Figure 3, Table 3). The goodness-of-fit of the SEM was as follows: χ^2^ = 76.59, *p* = 0.117, goodness of fit index: GFI = 0.800, adjusted goodness of fit index: AGFI = 0.712, comparative fit index: CFI = 0.946, and root mean square error of approximation: RMSEA = 0.070. All path coefficients were significant or showed a statistically significant tendency in this model. The test power of this model was calculated by performing a post hoc test using G*Power software. The number of main paths in the model was four; therefore, the setting predictors were four and the effect size was large (f^2^ = 0.35). The power of the test was 0.87, which was sufficient. The partial correlation test was conducted to confirm the absence of partial correlations in this model. Partial correlations between daily physical activity and cognitive function indexes controlled by the two-step test, which was the highest coefficient index in physical activity, were all not significant (Supplemental Table S1). Furthermore, partial correlations between indexes in physical function and QOL, which are controlled by Symbol Search, and partial correlations between indexes in cognitive function and happiness, which are controlled by QOL, were not significant (Supplemental Tables S2 and S3).

## 4. Discussion

This study reveals, for the first time, a series of axis from daily physical activity to happiness in older adults. In the axis of SEM, daily physical activity explains physical function, and physical function explains cognitive function. Cognitive function explains subjective mental health, happiness, and QOL (Figure 3, Table 3). The goodness-of-fit values of the SEM were GFI = 0.800, AGFI = 0.712, CFI = 0.946, and RMSEA = 0.070, and all path coefficients were significant or showed a significant tendency. Partial correlations were also calculated and no significant correlations were found (Supplemental Tables S1–S3). This suggests that the causal relationship shown in this study (Figure 3) is not due to any confounders included in this model. Table 2 shows that daily physical activity is correlated with many components of subjective mental health, cognitive function, and physical function. Poor “subjective” physical function revealed in GLFS-25 is related to the low subjective mental health and daily physical activity levels, not to the actual physical function level (Table 2, Figure 2A–C). In other words, feeling a physical impairment rather than having an actual physical disability may lead to low subjective mental health and daily physical activity levels.

Daily physical activity was shown as an initial structure in the present SEM; physical function was strongly associated with cognitive function (β = 0.50) (Figure 3). The inverse direction would have resulted in a lower coefficient and a lower goodness-of-fit model The SEM also clearly indicated a causality relationship between cognitive function and subjective mental health. Previous studies concerning the relationship between cognitive function and subjective mental health did not prove the direction of the relationship [18,19]. However, our study suggests that cognitive function produces better QOL and happiness levels. In the axis of the relationship, QOL preceded happiness within subjective mental health. The path coefficient linking QOL and happiness was the highest in the model (β = 0.76). Happiness was located at the final point in this model, and various factors in the lives of older adults were continuously connected to happiness. This suggests that QOL, cognitive function, physical function, and daily physical activity were all related to improved happiness.

To explain the causal relationship shown in this study, it is essential to consider the interaction between the brain and locomotor system. For the interaction between the brain and muscle, myokines and lactate play important roles. For example, cathepsin B and irisin are secreted from muscles by exercise, then BDNF is upregulated in the brain, resulting in neurogenesis in the hippocampus and promoting memory and other cognitive functions [28]. Lactate is also released into the blood and transferred to the hippocampus, especially during high-intensity exercise [29]. Thus, myokines could also be involved in the causal relationship shown in this study. However, the relationship between any myokines and happiness has not been reported to date, and future studies are needed.

Evidence of physical activity-induced happiness was previously reported as follows: a large-scale data analysis of 12,051 adults indicated that physical activity had a positive impact on happiness [30]. A comparison of a sedentary and exercise group showed that physical activity intervention slowed the decline in health-related QOL scores [16]. A large-scale health survey indicated that leisure-time physical activity reduced the odds of unhappiness [17]. In this study, we also indicated that happiness was influenced by physical activity. Very interestingly, we also found that cognitive function was an influential factor linking physical activity and happiness.

A survey study of 9761 older adults showed that high affective well-being may be associated with longer disability and chronic disease-free life expectancies [31]. A longitudinal cohort study of 9899 older adults revealed a positive association between enjoyment of life and survival [32]. The results strongly suggest that happiness is a critical factor for a long, healthy life. Based on the present study, it is possible to construct a strategy to achieve a healthy, long life by modifying daily physical activity. We propose that daily physical activity may become a useful and practical strategy to maintain functional and mental health, including well-being. If this strategy will be widely disseminated through organized community efforts, a long life and better well-being is expected in a high number of people. Although several previous studies on physical activity utilized exercise intervention with some intensity, the daily physical activity we focused on is a more feasible and sustainable intervention for individuals. According to a cohort study, 551 older adults whose frailty improved over two years had a higher amount of MVPA than those whose frailty worsened [33]. This suggests that enhancing the intensity of daily activity can improve frailty. This study showed that the promotion of daily physical activity is an important strategy to avoid physical frailty and may also help prevent mental frailty. We will perform intervention studies using different daily physical activity menus in the future.

The present study presented limitations. First, it was performed on healthy older adults. Therefore, it cannot be denied that other relationships exist in the case of people with disabilities and diseases. Second, the sample size in this study was small. Although the analysis was conducted to ensure enough power, a larger sample size would allow for more flexible modeling. Finally, this study recruited participants from a limited geographic area. Future studies may need to confirm whether locality affects the results.

## 5. Conclusions

This study clarified the interactive relationship as an axis starting from daily physical activity followed by physical function, cognitive function, QOL, and ultimately happiness in older adults. This means that happiness is influenced by physical and cognitive functions and social factors. This study suggests that upregulating daily physical activity, which is the initiating point of the axis, may improve physical and cognitive functions as well as mental health; this could protect and ameliorate physical, mental, and social frailties, and extends healthy life.

## Figures and Tables

**Table 1:** Demographic characteristics and means of evaluated scores.

	Mean	SD	95% CI		
Chraracteristics					
Gender (Male:Female)	24:21				
Age (years)	72.78	5.23	71.25	–	74.30
Education (years)	14.18	2.26	13.52	–	14.84
Subjective mental health					
Happiness (points)	3.98	0.54	3.82	–	4.14
QOL (points)	3.60	0.46	3.46	–	3.73
Cognitive function					
MMSE-J	28.29	1.41	27.88	–	28.70
WAIS-Ⅳ	87.42	11.97	83.92	–	90.92
Block design	11.58	2.79	10.76	–	12.39
Similarities	12.02	2.78	11.21	–	12.83
Digit Span	13.07	2.90	12.22	–	13.91
Matrix reasoning	13.04	3.10	12.14	–	13.95
Symbolic search	12.31	2.90	11.46	–	13.16
Comprehension	13.02	2.57	12.27	–	13.77
Coding	12.38	2.11	11.76	–	13.00
Physical function					
GLFS-25	5.90	5.40	4.32	–	7.48
5 m walk test (m/s)	2.35	0.52	2.20	–	2.50
2 step test (m/step)	1.29	0.15	1.25	–	1.34
Stand up test (points)	18.09	12.83	14.34	–	21.84
Daily physical activities					
Steps (steps/day)	6103.19	2859.59	5267.69	–	6938.69
Calore consumption (kcal/day)	134.76	117.84	100.33	–	169.19
MVPA(%)	1.44	1.48	1.01	–	1.88

Happiness: Oxford Happiness Questionnaire; QOL: WHOQOL26; MMSE-J: Mini-Mental State Examination-Japanese version; WAIS-IV: Wechsler Adult Intelligence Scale—4th ed.; GLFS-25: 25-Question Geriatric Locomotive Function Scale; and MVPA: Moderate-to-Vigorous Physical Activity.

**Table 2:** Correlates of all indicators of subjective mental health, cognitive function, physical function, and daily physical activity.

Indicator		Subjective Mental Health				Cognitive Function				Physical Function								Daily Physical Activities			
	Test	QOL		Happiness		MMSE−J		WAIS−Ⅳ		GLFS−25		5 m Walk		2−Step		Stand up		Calorie Consumption		Steps	
Subjective mental health	Happiness	0.70	**																		
Cognitive function	MMSE-J	0.11		−0.09																	
	WAIS-Ⅳ	0.14		0.00		0.43	**														
Physical function	GLFS-25	−0.39	**	−0.28	#	0.06		−0.13													
	5 m walk	0.08		0.12		0.10		0.34	*	−0.12											
	2-step	0.06		−0.01		0.51	**	0.21		−0.07		0.51	**								
	Stand up	0.25		0.04		0.30	*	0.27	#	−0.15		0.19		0.44	**						
Daily physical activities	Calorie consumption	0.15		0.09		0.25	#	0.25		−0.28	#	0.15		0.31	*	0.34	*				
	Steps	0.09		−0.08		0.18		0.08		−0.26	#	0.05		0.28	#	0.24		0.78	**		
	MVPA	0.26	#	0.08		0.28	#	0.28	#	−0.25	#	0.04		0.30	*	0.30	*	0.83	**	0.83	**

# *p* > 0.1, * *p* > 0.05, ** *p* > 0.01. Happiness: Oxford Happiness Questionnaire; QOL: WHOQOL26; MMSE-J: Mini-Mental State Examination-Japanese version; WAIS-IV: Wechsler Adult Intelligence Scale—4th ed.; GLFS-25: 25-Question Geriatric Locomotive Function Scale; and MVPA: Moderate-to-Vigorous Physical Activity.

**Table 3:** Details of path coefficients in Figure 3.

			*β*	*p*	
Between indicator index					
Daily physical activity	→	Physical function	0.29	0.095	#
Physical function	→	Cognitive function	0.50	0.026	*
Cognitive function	→	QOL	0.28	0.098	#
QOL	→	Happiness	0.76	0.000	***
Within indicator index					
Daily physical activity	→	MVPA	0.97	0.000	***
Daily physical activity	→	Steps	0.86	0.000	***
Daily physical activity	→	Calorie consumption	0.91	0.000	***
Physical function	→	2-step	0.85	0.000	***
Physical function	→	5 m walk	0.61	0.005	**
Physical function	→	Stand up	0.40	0.031	*
Cognitive function	→	Brock design	0.69	0.000	***
Cognitive function	→	Digit span	0.67	0.000	***
Cognitive function	→	Symbol Search	0.74	0.000	***
Cognitive function	→	Coding	0.72	0.000	***
Cognitive function	→	MMSE-J	0.50	0.004	**

Values of *β* show path coefficients. # *p* > 0.1, * *p* > 0.05, ** *p* > 0.01, *** *p* > 0.001. QOL: WHOQOL26; Happiness: Oxford Happiness Questionnaire; MVPA: Moderate-to-Vigorous Physical Activity; and MMSE-J: Mini-Mental State Examination-Japanese version.

## Data Availability

The data that support the findings of this study are not publicly available due to their containing information that could compromise the privacy of research participants but are available from the corresponding author.

## Supplementary Materials

The following supporting information can be downloaded at: https://www.mdpi.com/article/10.3390/ijerph20043016/s1. Table S1: Partial correlations between cognitive function and daily physical activities controlled by 2-step test; Table S2: Partial correlations between physical function and QOL controlled by Symbol Search; Table S3: Partial correlations between happiness and cognitive function controlled by QOL.
