# Supplementary material for: Causal Relationships between Daily Physical Activity, Physical Function, and Cognitive Function Ultimately Leading to Happiness
Source: Int J Environ Res Public Health. 2023 Feb 9;20(4):3016. doi: 10.3390/ijerph20043016 (PMC9958842; doi:10.3390/ijerph20043016)
Supplement: Supplementary file 1 [file ijerph-20-03016-s001.zip › ijerph-2153119-supplementary.pdf]

Supplemental Table S1. Partial correlations between cognitive function and daily physical activities controlled by 2-step test

| | | Cognitive function | | | | |
|---|---|---|---|---|---|---|
| | | Block Design | Digit Span | Symbol Search | Coding | MMSE-J |
| Controlled variable: 2 step test | Calorie consumption | -0.06 | -0.10 | 0.05 | -0.06 | 0.14 |
| | Steps | -0.08 | -0.17 | 0.02 | -0.23 | 0.31 |
| | MVPA | -0.18 | -0.22 | 0.03 | -0.21 | 0.11 |

MMSE-J: Mini Mental State Examination- Japanese version and MVPA: Moderate-to-Vigorous Physical Activity

Supplemental Table S2. Partial correlations between physical function and QOL controlled by Symbol Search

| | | Physical function | | |
|---|---|---|---|---|
| | | Stand up | 5 m walk | 2-step |
| Controlled variable: Symbol Search | QOL | 0.16 | 0.00 | 0.00 |

QOL: WHOQOL26

Supplemental Table S3. Partial correlations between happiness and cognitive function controlled by QOL

| | | Happiness |
|---|---|---|
| Controlled variable: QOL | Block Design | -0.07 |
| | Digit Span | -0.02 |
| | Symbol Search | -0.05 |
| | Coding | 0.10 |
| | MMSE-J | -0.22 |

Happiness: Oxford Happiness Questionnaire; QOL: WHOQOL26; MMSE-J: Mini Mental State Examination- Japanese version
